# Necrotizing Fasciitis Mimicking Interstitial Cystitis: An Atypical Clinical Presentation

**DOI:** 10.7759/cureus.68649

**Published:** 2024-09-04

**Authors:** Summer B Piwowarski, Michael W McGehee, Derek W White

**Affiliations:** 1 Surgery, Edward Via College of Osteopathic Medicine, Blacksburg, USA; 2 Family Medicine, Ballad Health, Abingdon, USA

**Keywords:** debridement fascitis, urinary tract infection complication, bladder ca, chronic interstitial cystitis, necrotizing fasciitis management, necrotizing fasciitis (nf), abdominal wall necrotizing fasciitis

## Abstract

Necrotizing fasciitis is a severe, life-threatening disease with a nonspecific clinical presentation, making it a challenging diagnosis. Early treatment with broad-spectrum antibiotics and surgical debridement is crucial to prevent rapid disease progression and poor outcomes. Given its high mortality rate and ambiguous presentation, maintaining a high index of suspicion for necrotizing fasciitis is essential.

In this case, a 60-year-old woman presented to her gynecologist with urinary tract infection symptoms of frequency, hematuria, and suprapubic pain, with a year-long history of night sweats, hematuria, dysuria, and incomplete voiding. Although initially treated with outpatient antibiotics, she returned to the emergency department one day later with severe lower abdominal pain, overlying erythema, and a high fever. Abdominal imaging revealed extensive cellulitis. Upon the development of rapidly expanding erythema and crepitus, there was concern for necrotizing fasciitis. The patient received immediate treatment with broad-spectrum antibiotics and underwent urgent surgical debridement. While she showed clinical improvement in the following days, laboratory studies revealed profound hypercalcemia, anemia, and persistent leukocytosis. Additional testing ultimately led to the diagnosis of advanced bladder cancer.

This case underscores the importance of prompt recognition and treatment of necrotizing fasciitis. It also highlights the influence of confirmation and availability biases, which can lead to overlooking symptoms that may indicate more serious underlying conditions. As medical professionals, it is crucial to remain vigilant and not disregard seemingly insignificant symptoms, as they could be indicative of life-threatening diagnoses.

## Introduction

Necrotizing fasciitis is a rare, severe, life-threatening soft tissue infection that is associated with a high morbidity and mortality rate if not treated promptly and aggressively. Necrotizing soft tissue infections are characterized by tissue inflammation, gas formation, and fulminant destruction [1,2]. They can progress rapidly and are difficult to diagnose, making it necessary to keep a high index of suspicion.

Necrotizing soft tissue infection is a broad term that encompasses necrotizing cellulitis (subcutaneous tissue), necrotizing fasciitis (fascia), and necrotizing myositis (muscle), depending on the location of tissue involved [3-5]. Necrotizing fasciitis most commonly involves the superficial fascial layer due to its relatively poor blood supply, with frequent extension into the overlying subcutaneous fat. Muscle tissue is less commonly involved due to its ample blood supply [6]. Often, the overlying skin will appear unaffected, making it a difficult diagnosis clinically.

Necrotizing fasciitis is caused by a severe bacterial infection, in lay terminology referred to as “flesh-eating bacteria." It is most often caused by multiple organisms (polymicrobial, type I), but it can also be due to a single organism (monomicrobial, type II) [7-9]. In polymicrobial infections, anaerobic species (e.g., Clostridium perfringens) with Enterobacteriaceae (e.g., Klebsiella pneumoniae) and/or streptococci (e.g., Streptococcal pyogenes) are often found on cultures [6,10,11]. Monomicrobial infections are more likely to be either Streptococcal pyogenes or Staphylococcus aureus [12,13]. Streptococcus pyogenes, otherwise known as group A streptococcus (GAS), is the most commonly identified cause of necrotizing fasciitis. According to cases tracked by the CDC, there are approximately 700 to 1,150 cases per year of necrotizing fasciitis caused by GAS [14].

The overall incidence of necrotizing fasciitis is around 0.3 per 100,000 population [3]. Trauma, resulting in a breach in the skin’s protective barrier, is the most common etiology of necrotizing fasciitis. Unlike monomicrobial (type II) necrotizing infections, which occur at any age with no comorbidities [6], polymicrobial infections are highly associated with elderly immunocompromised patients [3,6,10,14]. This is especially true for diabetics and those with peripheral vascular disease [6,11]. Other risk factors include recent surgery, IV drug use, immunosuppressive drug use, malignancy, obesity, alcoholism, and childbirth [3,6,10,14].

Necrotizing fasciitis most often occurs acutely, over hours, and has a predisposition to affect the lower extremities more often than the upper [1,2]. Infections in the extremities are associated with a lower mortality rate compared to infections in the trunk or perineum [15,16]. This is because extremity amputation above the area of infection can provide a definitive cessation of bacterial spread, although this is considered a last-resort treatment for the uncontrollable spread of infection. Typical symptoms of necrotizing fasciitis include high fever (102-105°F), tachycardia, hypotension, and severe pain [3,4,17-19]. Patients may also have nonspecific symptoms such as malaise, myalgia, diarrhea, and anorexia. On exam, the patient may have poorly demarcated erythema with edema that extends beyond the margins, warmth, crepitus, and less commonly, skin bullae, necrosis, or ecchymosis [3,4,17-19]. Most of the signs and symptoms of necrotizing fasciitis are nonspecific; therefore, keeping a high index of suspicion is crucial for rapid diagnosis [3].

Diagnostic imaging is utilized in cases with a moderate to low suspicion of necrotizing fasciitis when surgical intervention is not immediately indicated. A computed tomography (CT) scan is the most commonly used imaging modality for the evaluation of suspected necrotizing fasciitis. The most specific CT finding is gas within fluid collections tracking along fascial planes [20]. Audible crepitus to palpation during physical exam is only present in around 38% of patients and is more likely in polymicrobial infections [6]. Often, the subcutaneous tissues will just be firm and indurated [4,19].

Laboratory markers of necrotizing fasciitis are nonspecific and may include leukocytosis (elevated white blood cell count), elevated creatinine, elevated lactate, hyponatremia, and/or elevated markers of inflammation such as C-reactive protein and erythrocyte sedimentation rate. Elevations in creatinine kinase (CK) or aspartate aminotransferase (AST) may present if the infection has spread to the deep muscle or fascial layers [3]. The Laboratory Risk Indicator for Necrotizing Fasciitis (LRINEC) is a scoring system comprised of six laboratory values that can be used to distinguish necrotizing fasciitis from other soft tissue infections as well as provide prognostic indications [21-23]. The values incorporated into the score include C-reactive protein, hemoglobin, total leucocyte count, serum sodium, serum creatinine, and blood glucose levels. A LRINEC score of greater than six has a positive predictive value of 92% for necrotizing fasciitis and a negative predictive value of 96% [21]. The LRINEC score can be used to support diagnosis and subsequent surgical intervention in patients with borderline or ambiguous clinical presentation [21]. Because the laboratory findings are non-predictive and mainly indicate some infectious process, diagnosis or exclusion is not made by the presence or absence of these markers [21-23].

If left untreated over several days, the tissues will experience rapid breakdown. The overlying skin will change color from red to blue-gray with eventual breakdown and bullae formation. The nerves and blood supply will be cut off, resulting in diminished pain sensation and gangrene formation. The outcomes of this could be loss of limb, irreversible damage, and death [1,2]. The mortality rates, even with optimal therapy, range from 14%-34% [1,2,6]. Mortality in cases of necrotizing fasciitis is due to overwhelming septic shock and organ failure and is positively correlated with time to surgical intervention [24].

A definitive diagnosis of necrotizing fasciitis is established surgically through a physical examination of the tissues involved [3]. Therefore, prompt surgical debridement is the mainstay of both diagnosis and treatment. Findings of edematous gray fascia, non-purulent thin gray discharge known as “dishwater discharge," and easy separation of tissue planes by blunt dissection are diagnostic [4,19]. Radiographic studies can be useful in the diagnosis. The best initial imaging test is a CT scan, as it may show subcutaneous gas, which is highly specific for necrotizing fasciitis [10,25,26]. However, diagnostic workup such as imaging or establishing antibiotic treatment should not delay early intervention via surgery [1]. The mortality rate of necrotizing fasciitis that is treated with antibiotics alone is close to 100% [10]. If early surgical intervention is performed, ideally within six hours of admission, the mortality significantly improves [18].

There is also some evidence to support the use of hyperbaric oxygen therapy as an adjunctive treatment for necrotizing fasciitis [27]. The theory behind this modality is that increasing oxygenation of infected tissue helps salvage critically ischemic tissue, thus reducing the number of surgical debridements required. It is also suggested that hyperoxia potentiates white blood cell killing, improves antibiotic effectiveness, and reduces inflammation, which can lead to better outcomes. However, it should be noted that no high-quality trials have been performed, and randomized evidence is insufficient to support or refute the use of hyperbaric oxygen as an adjunctive treatment for necrotizing fasciitis [27].

With the life-threatening nature of this disease and ambiguous clinical presentation, clinicians must have a firm understanding of the various symptoms and abstruse presentations so that a swift diagnosis can be made and life may be saved.

## Case presentation

The patient was a 60-year-old woman with a past surgical history of cesarean section and hysterectomy, a 30-pack-year smoking history, and opioid dependence on suboxone. The patient's only other medications included fluticasone and cetirizine for allergies. She had medication allergies to azithromycin and prednisone. She presented to her gynecologist with complaints of a three-month history of painful urination and hematuria. She reported waking up frequently, about four to five times per night, to urinate. However, she denied urinary incontinence. She described decreased urine volume with incomplete voiding of the bladder. She also had painful cramping of the bladder during urination. She had been having consistent night sweats for the first few hours of the night, so severe she had to change her clothes. She also would have an episode of chills about once a week. She reported feeling fatigued and attributed this to her poor sleep. She described a life-long history of urinary tract infections (UTI). However, she said that this time did not feel like any UTI she has had in the past. She tried limiting fluid intake in the evening, but that did not reduce the night-time urination frequency. She had been taking six ibuprofens per day for the pain. On exam, the patient had severe suprapubic tenderness with significant guarding but no costovertebral angle tenderness or flank pain. The patient’s urine analysis was negative for nitrates but positive for leukocyte esterase with a large amount of blood. The patient was counseled by the gynecologist regarding several features concerning hemorrhagic interstitial cystitis. The patient was treated with outpatient nitrofurantoin, and urine cultures were sent. She had an appointment with the urologist the following day, which she was counseled to keep. She was told to follow up with the gynecologist in three to four weeks.

The patient presented to the emergency room one day later for right lower quadrant abdominal pain. On exam, the patient had a well-demarcated area of erythema and induration in the right lower quadrant with a 1-2 cm area of ecchymosis. No fluctuance mass, subcutaneous emphysema, or drainage was observed. The patient denied fever, chills, nausea, vomiting, or diarrhea. The patient did endorse dysuria and hematuria. She denied any injection drug use in the area. The patient’s temperature was 98.5 °F, with a heart rate (HR) of 79 beats per minute (bpm), blood pressure (BP) of 99/57 mmHg, respiration rate (RR) of 18, and oxygen saturation of 92% on room air. The patient had marked neutrophil-predominant leukocytosis of 18.2 K/uL. Her hemoglobin (Hb) was 10.4 g/dL, and her platelet count was 303,000 platelets per microliter. Kidney function was consistent with previous measurements, with a creatinine of 0.9 mg/dL and a blood urea nitrogen (BUN) of 19 mg/dL. Serum sodium was 138 mmol/L, and blood glucose was 117 mg/dL. Urinalysis showed large blood, leukocyte esterase positive, nitrate negative, 3+ protein, with >50 red blood cells (RBC), >50 white blood cells (WBC), and many bacteria. Her lactate was normal, 1.3 mmol/L, and total protein was low-normal, 6.0 g/dL (Table 1).

**Table 1:** Referance ranges for studied parameters The patient's value for each parameter was studied for comparison to the standard. The reference range corresponds to the levels considered normal for women aged 60 years old.

Parameter Studied	Patient’s Value	Reference Range
Temperature (Fahrenheit (°F))	98.5	97.7–99.5
Heart rate (beats per minute (bpm))	79	60–100
Blood pressure (millimeters of mercury (mmHg))	99/57	90–120/60–80
Mean arterial pressure (millimeters of mercury (mmHg))	65	65–100
Respiration rate (respirations per minute)	18	12–20
Oxygen saturation (SpO2)	92%	95–100%
Leukocyte count (cells per microliter (cells/µL))	18.2	4,500–11,000
Leukocyte count, second measurement	18.2	4,500–11,000
Leukocyte count, third measurement	13.6	4,500–11,000
Leukocyte count, fourth measurement	21.4	4,500–11,000
Hemoglobin (Hb) (gram per deciliter (g/dL))	10.4	12.0–15.5
Hemoglobin, second measurement	8.9	12.0–15.5
Hemoglobin, third measurement	8.9	12.0–15.5
Hemoglobin, fourth measurement	9.3	12.0–15.5
Platelet count (platelets per microliter (plt/µL))	303,000	150,000–450,000
Creatinine (milligram per deciliter (mg/dL))	0.9	0.6–1.1
Blood urea nitrogen (BUN) (milligram per deciliter (mg/dL))	19	6–22
Serum sodium (millimole per liter (mmol/L))	138	135–145
Blood glucose (milligram per deciliter (mg/dL))	117	70–100
Lactate (millimole per liter (mmol/L))	1.3	<2
Total protein (gram per deciliter (g/dL))	6.0	6.0–8.3
Serum calcium (milligram per deciliter (mg/dL))	14.6	8.5–10.5

The patient was given one dose of vancomycin and admitted to the hospital. To maintain oxygenation and blood pressure, the patient was intubated and given mechanical ventilation as well as intravenous (IV) norepinephrine drip with a mean arterial pressure goal of 65 mmHg (Table 1). The patient was treated with IV clindamycin, piperacillin-tazobactam, and vancomycin.

**Figure 1:**
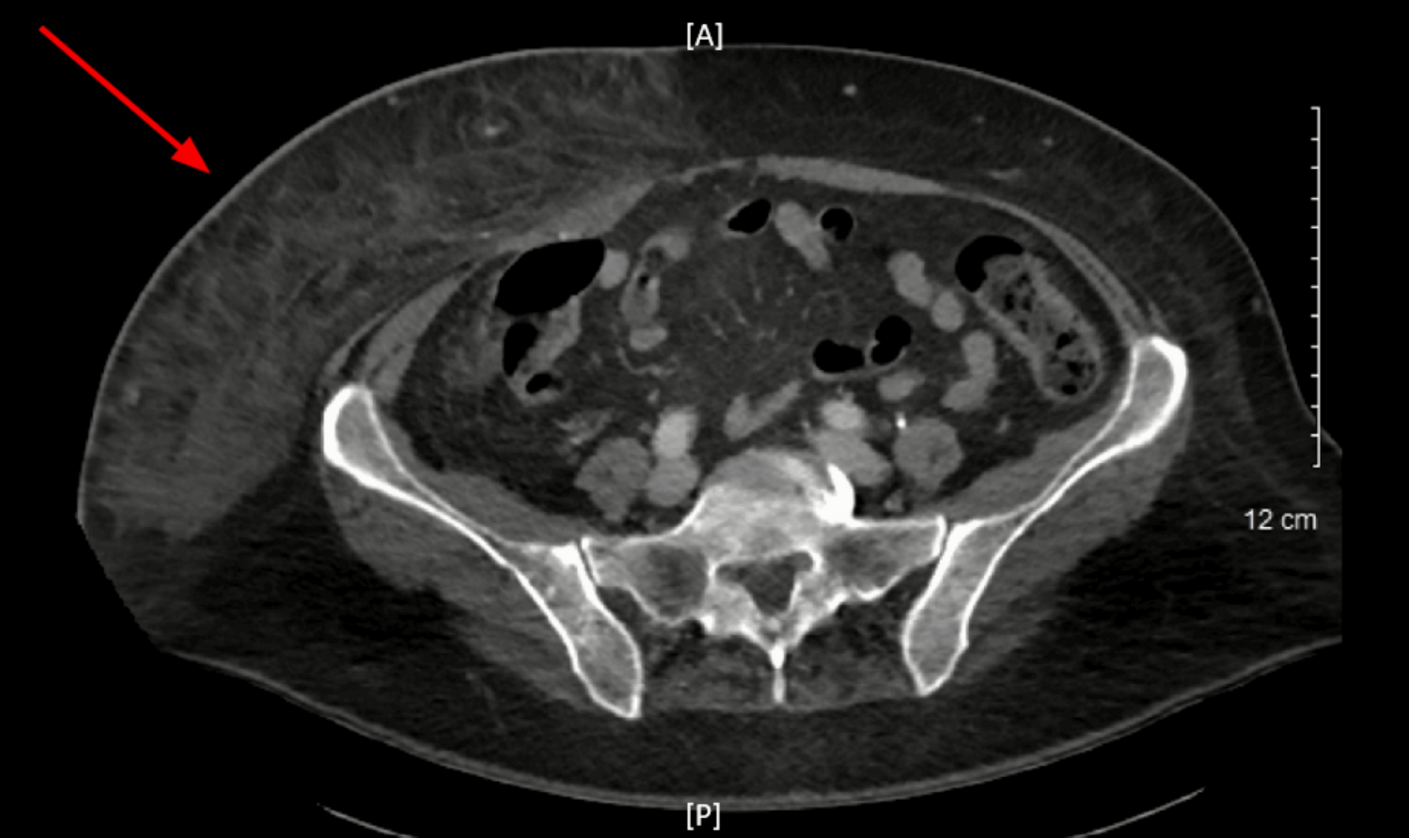
Axial computed tomography scan of the abdomen The red arrow shows skin thickening with extensive subcutaneous fat stranding in the right lower quadrant. These findings correlate to an area of fascial infection assisting with the diagnosis of necrotizing fasciitis. A: anterior; P: posterior

**Figure 2:**
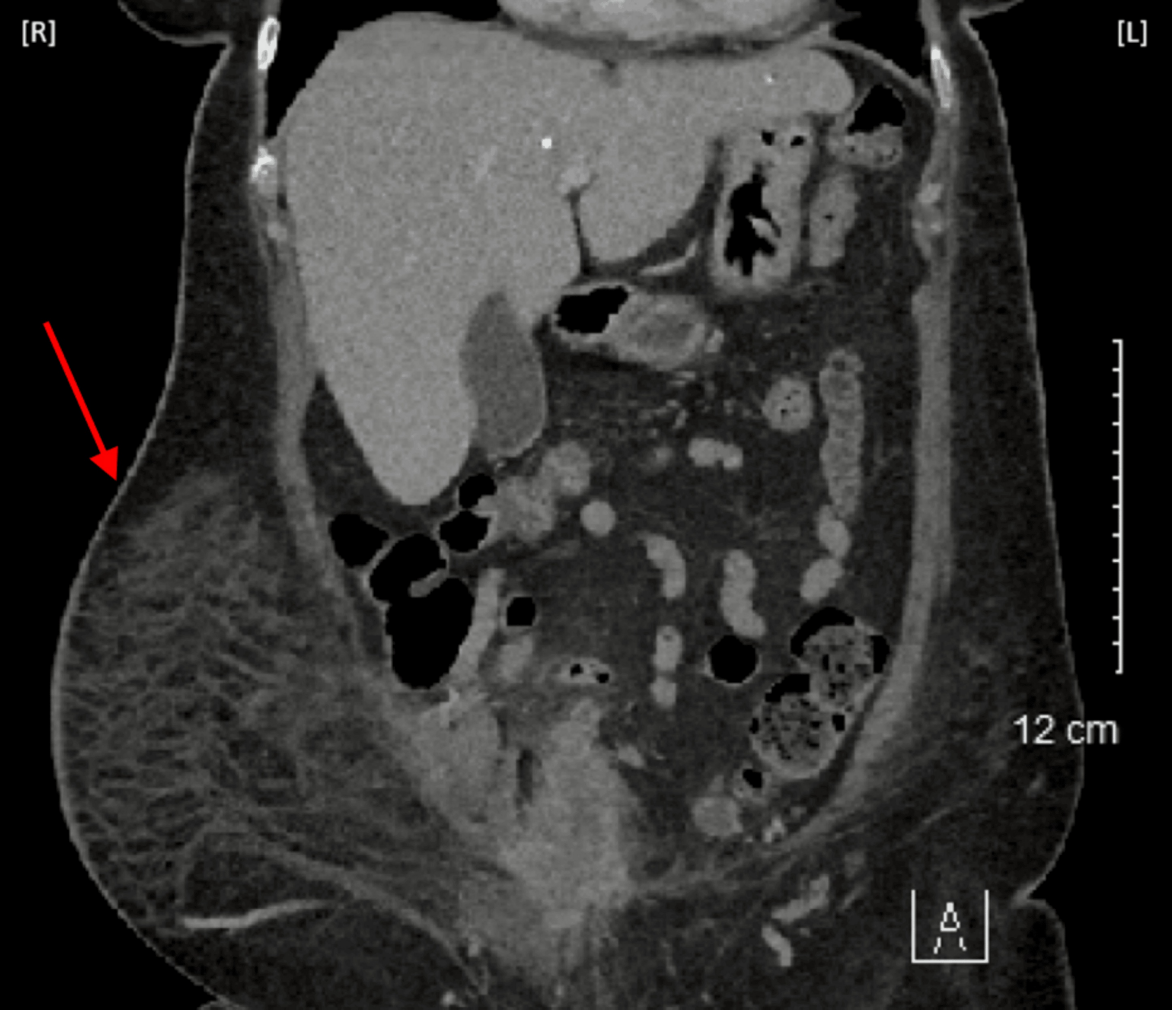
Coronal computed tomography scan of the abdomen and pelvis The red arrow identifies skin thickening with extensive subcutaneous fat stranding in the right lower quadrant of the abdomen. These findings correlate to an area of infection assisting in the diagnosis of necrotizing fasciitis. R: right; L: left

**Figure 3:**
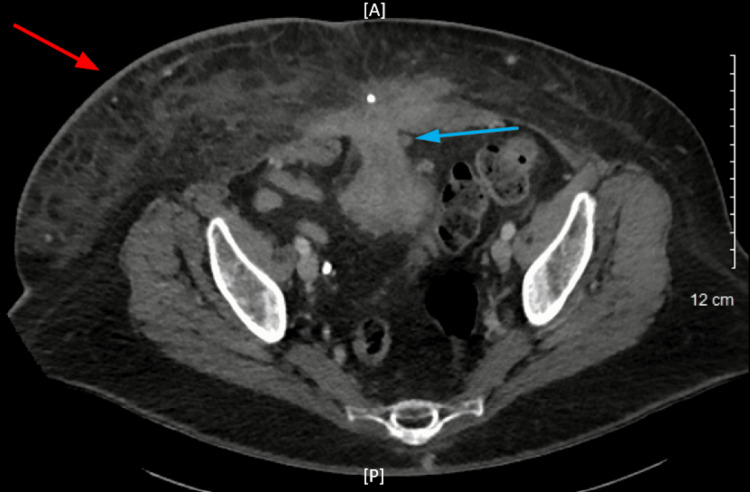
Axial computed tomography scan of the abdomen The red arrow shows skin thickening with extensive subcutaneous fat stranding in the right lower quadrant which correlates to the area of infection. The blue arrow points to the bladder herniation through midline abdominal wall defect. A: anterior; P: posterior

Within a few hours, the erythema rapidly doubled in size to 15 cm, and a small area of necrosis was observed with underlying crepitus. The right lower abdominal quadrant was rigid and very tender upon palpation. Hypoactive bowel sounds were noted. Surgery was immediately notified. Computed tomography (CT) imaging of the abdomen was obtained. The CT scan showed skin thickening with extensive subcutaneous fat stranding in the right lower quadrant extending bilaterally in the inferior aspect of the abdominal pannus, consistent with cellulitis (Figure 1, 2). There was a recurrent midline ventral hernia in the lower anterior abdominal wall, and a portion of the urinary bladder protruded through the defect (Figure 3). Also observed was generalized irregular mural thickening and trabeculation of the urinary bladder wall, noted to be likely a chronic cystitis and/or possible neoplastic infiltration.

**Figure 4:**
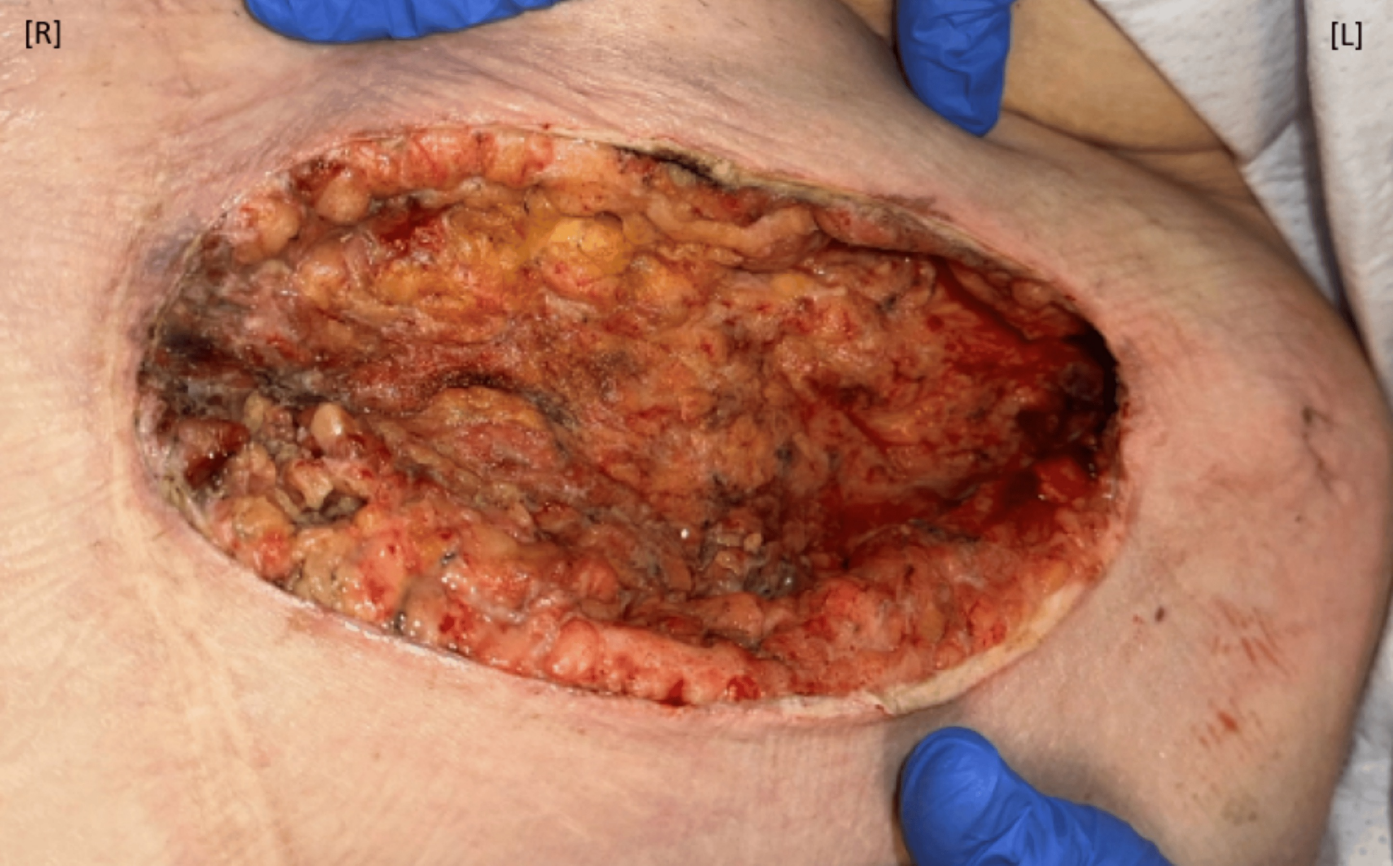
Wound after first surgical debridement. The necrotized abdominal tissues were removed via surgical debridment creating a wound that did not go past midline. R: right; L: left

After the concerning CT results, the patient was quickly brought to the operating room (OR). The patient was placed under general anesthesia and underwent excisional debridement of the necrotizing fasciitis using blunt, sharp electrocautery dissection with a hemostatic knife and a Bovie. The dissection tracked from the midline to the right iliac crest, and excisional debridement involved the fascia but did not go intra-abdominally (Figure 4). Hemostasis was achieved using Bovie electrocautery and 3-0 Vicryl (Ethicon, Inc. Raritan, NJ). The area was then irrigated using Puracyn (Innovacyn, Inc. Rialto, California). The wound was packed to remain open, and a wound vacuum-assisted closure (VAC) was placed. The patient tolerated the procedure well. Follow-up surgical debridement was planned for 48 hours later. The patient remained sedated and intubated in the intensive care unit (ICU).

The following day, the wound VAC was removed, and scant bleeding was observed, but no further purulent drainage or obvious necrosis was noted. The wound was irrigated with Puracyn and repacked. The patient's hemoglobin had dropped to 8.9 g/dL, and her leukocytes remained elevated to 18.2 K/uL (Table 1).

**Figure 5:**
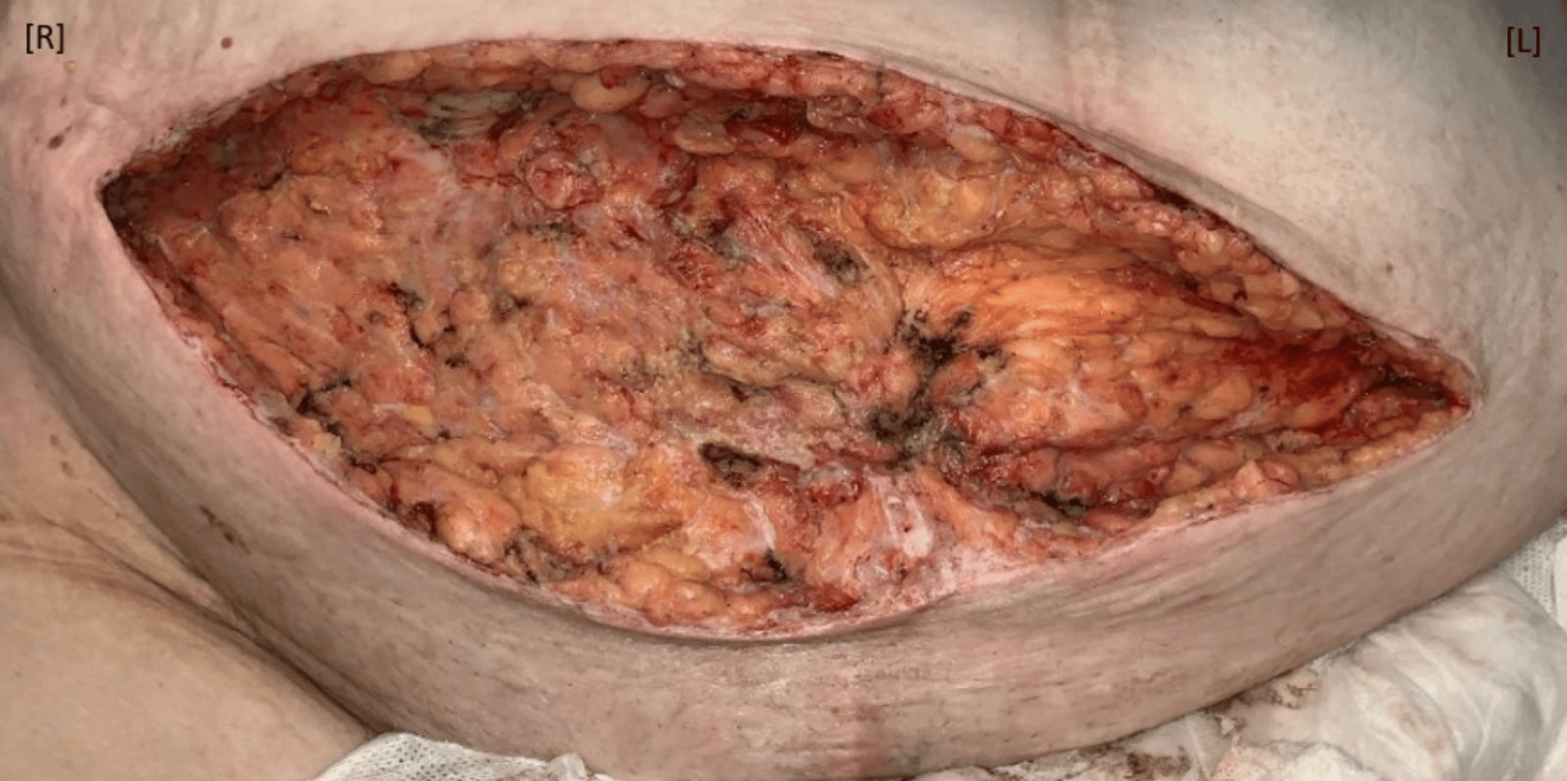
Wound after second surgical debridement. The remaining necrotized abdominal tissues were removed via a second surgical debridment creating a wound that went well past midline. R: right; L: left

The 48-hour follow-up debridement took place under general anesthesia in the operating room (OR) using similar techniques. A large abscess filled with pus was noted near the midline. Extending the margins of the original incision, the abscess was drained, and samples were taken for culture. The wound was irrigated with Puracyn. Following the second debridement, the wound measured 30 cm x 10 cm x 4 cm (Figure 5).

The next day, the patient was taken off sedation and ventilation. Wound care replaced the wound VAC. The patient’s leukocytes started to normalize, with a white blood cell count of 13.6 K/uL, but her hemoglobin remained low at 8.9 g/dL (Table 1). Her blood pressure was improving, so the patient was taken off IV norepinephrine and given an IV dopamine drip with a mean arterial pressure goal of 65 mmHg. The patient also had a fecal management system and Foley catheter in place.

Several days later, urine and wound gram stain and culture results returned. The urine showed growth of >100,000 Escherichia coli. The wound gram stain showed multiple anaerobic bacteria present, but the culture had no growth. No blood cultures were obtained during admission due to unknown circumstances. With a continuation of the antibiotics, the wound began to heal. About one week later, granulation tissue was observed, and there were no areas of necrosis.

**Figure 6:**
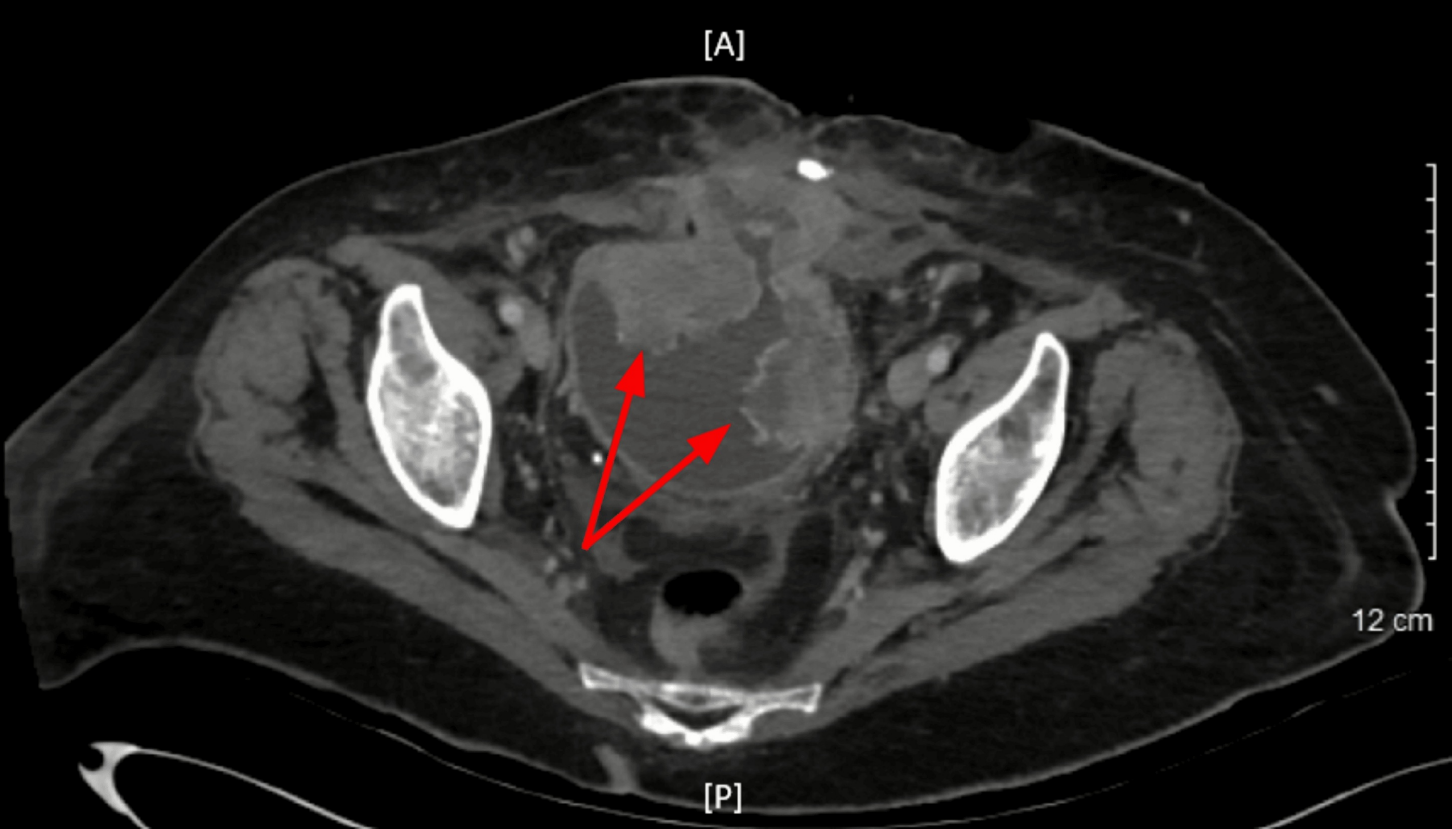
Axial computed tomography scan of the abdomen and pelvis The red arrows show an infiltrative mass involving the anterior and left lateral bladder walls extending into the herniated portion of the urinary bladder. This mass was later diagnosed as high-grade urothelial carcinoma. A: anterior; P: posterior

Over the next weeks, the patient began to have worsening leukocytosis, with white blood cell count increasing to 21.4 K/uL. She maintained a persistent normocytic anemia with a hemoglobin of 9.3 g/dL that did not resolve with supplemental iron (Table 1). However, the patient reported feeling better and remained afebrile with no clinical signs of worsening infection. She was treated with empiric oral amoxicillin and clavulanate (Augmentin, Jackson Healthcare, Bristol, Tennessee) and trimethoprim/sulfamethoxazole (Bactrim, Major Pharmaceuticals, Livonia, MI). A repeat urine culture grew extended-spectrum beta-lactamase (ESBL) Klebsiella pneumonia and Candida albicans. Antibiotics were changed to IV meropenem and empiric fluconazole. The patient's laboratory studies showed worsening hypercalcemia, reaching a level of 14.6 mg/dL (Table 1). With low vitamin D levels and low parathyroid hormone, this elevated calcium level was concerning for malignancy. A parathyroid hormone-related peptide test came back significantly elevated. A repeat CT showed extensive infiltrative bladder mass (Figure 6), which was confirmed by a cystogram. Urine cytology revealed findings suggestive of high-grade urothelial carcinoma. After one month in the hospital, the patient was discharged to hospice with palliative care.

## Discussion

This case is significant in two ways. First, necrotizing fasciitis is a rare disease with a nonspecific presentation. However, without early detection and rapid management, it is often fatal. There are few described cases in the literature of necrotizing fasciitis, and none in which the presentation started with nonspecific interstitial cystitis-like symptoms. With its ambiguous presentation, it is important for clinicians to have a comprehensive understanding of various ways in which necrotizing fasciitis can present. Second, this case highlights the effects of confirmation bias, which focuses on indicators that confirm an initial diagnosis while ignoring negative signs or findings that suggest a different diagnosis, and availability bias, which refers to reliance on commonly seen diagnoses instead of the careful assessment of data, on the medical decision-making process.

In this case, the patient presented to her gynecologist with common symptoms of frequency, hematuria, and dysuria. Her urinalysis confirmed a readily available and commonly seen diagnosis of urinary tract infection. However, the more concerning features of year-long night sweats, hematuria, dysuria, and incomplete voiding are not typical of a urinary tract infection. It is difficult to determine how these were taken into consideration in the initial diagnosis. The patient was seeing a urologist the following day, so these concerns may have been set aside for attention by the specialist. While difficult to determine after the fact, a more thorough investigation of these symptoms may have led to an earlier diagnosis and treatment.

When the patient presented to the hospital, the diagnosis of necrotizing fasciitis was delayed due to her nonspecific presentation. She had an acute presentation of severe lower abdominal pain with a recently diagnosed urinary tract infection. She had a well-demarcated area of erythema and induration in the right lower quadrant with overlying ecchymosis, but no subcutaneous emphysema or frank gangrene that would be diagnostic of necrotizing fasciitis. The patient was also afebrile and had relatively stable vital signs, with a heart rate of 70 bpm and blood pressure of 99/57 mmHg. Her marked leukocytosis and low hemoglobin were concerning; however, the other laboratory values that would be indicative of a necrotizing infection were normal (Table 1).

According to the Laboratory Risk Indicator for Necrotizing Fasciitis (LRINEC) score, she had a small likelihood of necrotizing fasciitis. A score greater than six predicts there is a 92% likelihood of necrotizing fasciitis infection. With a normal serum sodium, normal serum creatinine, and normal blood glucose level, the elevated white blood cell count and low hemoglobin alone would have made her LRINEC score three. However, this is assuming a normal serum C-reactive protein, a marker of inflammation, which was not measured on admission. Assuming an elevated C-reactive protein, her score would be seven, which is an intermediate risk for necrotizing infection. This highlights the importance of having cases in the literature so that physicians can improve their understanding of the criteria, the proper tests may be ordered, and life-threatening diagnoses are not missed.

Due to the patient’s nonspecific presentation, she was admitted to the hospital, treated with antibiotics, and received CT imaging. According to guidelines, these should be secondary to immediate surgical debridement for necrotizing fasciitis, which is both diagnostic and therapeutic. However, these guidelines are established for situations in which there is a high index of suspicion for necrotizing fasciitis. With her mild presentation, the likelihood of necrotizing fasciitis was intermediate to low. Without strong evidence to support necrotizing fasciitis, these guidelines should be balanced with the costliness and high risk of an operation. Nonetheless, keeping a low threshold for surgical treatment is important, as delaying the operation may necessitate larger margins of debridement and could prolong the healing course. Therefore, it is crucial to have abundant cases in the literature of various necrotizing fasciitis presentations so that preliminary diagnoses can be made quickly and treatment may be prompt.

After a month in the hospital with persistent leukocytosis, anemia, and hypercalcemia, despite aggressive treatment, the patient was diagnosed with bladder cancer. The CT scan she received upon admission suggested there were changes to the bladder wall, either due to chronic cystitis or malignancy. The CT also showed bladder herniation through the ventral abdominal wall. This begs the question of whether the severity of the patient’s infection was associated with her underlying malignancy or significant herniation. Further investigations should be made into whether high-grade urothelial carcinoma causes some predisposition to necrotizing fasciitis or if protrusion of the bladder into the abdominal fascia creates a susceptibility to a necrotizing infection.

## Conclusions

This case offers a unique examination of necrotizing fasciitis, particularly due to its atypical and non-specific presentation. Initially, the patient displayed symptoms predominantly associated with genitourinary issues, which led to significant diagnostic delays. This delay in recognizing the severity of necrotizing fasciitis impeded the timely administration of critical treatment, which is essential for resolving this life-threatening infection.

The study delivers crucial insights into the clinical manifestations of necrotizing fasciitis, emphasizing the need for a thorough differential diagnosis. It underscores that clinicians must remain vigilant and consider a broad spectrum of potential diagnoses rather than being swayed by more easily confirmed or common conditions. Such an approach is vital to avoid diagnostic overshadowing and to ensure that rare but severe conditions like necrotizing fasciitis are identified and managed promptly.

Moreover, the study highlights the imperative for swift diagnosis and intervention, given the rapid progression and high morbidity associated with necrotizing fasciitis. This urgency is compounded in patients with underlying comorbidities, which can further complicate the clinical picture and necessitate an even more prompt and aggressive treatment strategy.
